# Comparative assessment of the impact of iron deficiency on HbA1c accuracy in non-anaemic individuals with type 2 diabetes: A secondary data analysis

**DOI:** 10.1371/journal.pone.0323034

**Published:** 2026-02-02

**Authors:** Rogers John Mukasa, Nathan Mubiru, Isaac Sekitoleko, Ronald Makanga, Hubert Nkabura, Terry Ongaria, Viola Mugamba, Wisdom Nakanga, Moffat J. Nyirenda, Anxious J. Niwaha

**Affiliations:** 1 Medical Research Council/ Uganda Virus Research Institute and LSHTM Uganda Research Unit, Entebbe, Uganda; 2 Department of non-communicable diseases Epidemiology, London School of Hygiene and Tropical Medicine (LSHTM), London, United Kingdom; Kintampo Health Research Centre, GHANA

## Abstract

**Purpose:**

Results from a few studies have been conflicting regarding whether iron deficiency affects HbA1c reliability, and the mechanisms by which iron might influence HbA1c are not fully understood. We aimed to compare the relationship between HbA1c and average glucose levels measured by continuous glucose monitoring, retrospectively, in iron-replete and iron-deplete states among non-anemic type 2 diabetes mellitus (T2DM) patients.

**Methods:**

We compared the differences in HbA1c between iron-replete and iron-deplete groups using the Chi-square test for categorical data and the Mann-Whitney U test for continuous data. We also evaluated the correlation between HbA1c and mean plasma glucose for both iron-replete and iron-deplete individuals using Pearson’s correlation and linear regression.

**Results:**

A total of 146 of the 213 participants screened had complete data and were included in the final analysis. 43 out of 146 (29.5%) had iron deficiency, and 103 were iron-replete. No significant difference was observed in HbA1c levels between iron-replete and iron-deplete individuals: 69 (51.0, 85.0) vs 62 (46.0, 83.0) mmol/mol, P = 0.291). There was a strong positive correlation between HbA1c and mean plasma glucose concentration for both iron-replete and iron-deplete individuals (Pearson Correlation coefficient: 0.88 (0.83–0.92) and 0.93 (0.88–0.98), respectively).

**Conclusions:**

HbA1c correlates well with mean blood glucose even in the iron-deplete state amongst non-anaemic T2DM individuals. However, larger studies are needed to confirm these findings, particularly at screening and diagnostic thresholds.

## Introduction

Diabetes mellitus (DM) is a significant global health problem, currently affecting approximately 463 million individuals globally [1]. Of these, 336 million (approximately 4/5 of the world’s diabetic population) reside in low and middle-income countries (LMIC) [2]. Effective diabetes monitoring is essential for the appropriate titration of medication, enabling optimal glycaemic control and prevention of vascular complications.

Glycated haemoglobin (HbA1c) is widely recognized as the gold standard for monitoring glycaemic control [3]. However, it is increasingly evident that HbA1c can be influenced by non-glycaemic factors, potentially undermining its reliability as an accurate measure of long-term glycaemic exposure [4,5]. Iron deficiency (ID), the most common micronutrient deficiency worldwide [6] has been identified as one of the factors that may alter HbA1c levels. The proposed mechanisms underlying the interaction between ID and HbA1c remain poorly understood, but include: alteration of the haemoglobin globin chain quaternary structure in iron deficiency, leading to rapid glycation, increased average erythrocyte life span in iron deficiency, hence increased HbA1c, and increased glycation of the proline terminal through peroxidation by the reduction of the anti-oxidant capacity of iron-containing enzymes in the iron-deficient state [7,8].

Despite a broad consensus that iron status can biologically influence HbA1c measurements, the existing literature on the relationship between ID and HbA1c, remains inconsistent and methodologically limited. Most previous studies have been constrained by small sample sizes and inadequate control for important confounding such as anaemia and hemoglobinopathies. As a result, findings across studies are contradictory, and fail to fully explain the nature of the association. For example, while some studies report elevated HbA1c levels in individuals with iron deficiency, others report reduced levels or no significant difference between the two groups [9]. These inconsistencies highlight a critical gap in understanding how iron deficiency affects the interpretation of HbA1c as a marker of long-term glycaemic control.

Understanding the relationship between HbA1c and mean glucose levels in patients with type 2 diabetes and ID is particularly important, especially in LMICs, where both diabetes and iron deficiency are highly prevalent, and routine screening for ID among patients with diabetes is uncommon. Our study aimed to examine the impact of ID on HbA1c and explore the relationship between HbA1c and mean glucose levels, as measured by continuous glucose monitoring (CGM), in both iron-deplete and iron-replete states among non-anaemic patients with type 2 diabetes participating in the OPTIMAL study.

### Study methods

This was a retrospective secondary data analysis of the OPTIMAL observational study, a multicentre study conducted between 01^st^ August 2019 and 27^th^ February 2020, at two healthcare facilities: St. Francis Hospital Nsambya and Masaka Regional Referral Hospital in Uganda. The study enrolled adults aged 18 years and above with a clinical diagnosis of type 2 diabetes mellitus of more than 12 months’ duration. Eligible participants had not required insulin therapy during the first year following diagnosis, had no changes in their glucose-lowering therapy within the three months prior to enrolment. We excluded pregnant women, critically ill patients, those with anaemia, renal impairment, and haemoglobinopathies. All participants provided written consent before their involvement in the study.

A detailed description of the study procedures has been described elsewhere [10]. In summary, participants attended three study visits. During Visit 1, they arrived in a non-fasted state, and clinical and socio-demographic data, including age and sex, were collected using a standardized structured questionnaire. Trained research nurses obtained anthropometric measurements, recording weight in kilograms and height in meters. A non-fasting random blood sample (within 5 hours of a meal) was taken for laboratory analysis, which included assessments of Complete Blood Count (CBC), HbA1c (mmol/mol), glucose (mmol/L), and iron levels (µg/L). Continuous glucose monitoring was initiated using the Freestyle Libre Pro Flash Glucose Monitoring System (Abbott Laboratories, Illinois, USA), which continuously records interstitial glucose levels every 15 minutes for 14 days.

During visit 2, which occurred between days 7 and 10 of continuous glucose monitoring, participants returned in a fasted state (at least 8 hours after their last meal) for fasting glucose measurement. During visit 3, held between days 12 and 14 from the baseline visit, participants returned in a non-fasted state (within 5 hours of a meal) for CGM data download and venous blood sample collection for HbA1c measurement.

### Laboratory analysis

Plasma glucose, HbA1c, renal function, and full blood count were analysed at the Medical Research Council and London School of Hygiene and Tropical Medicine/ Uganda Virus Research Institute (MRC & LSHTM/ UVRI) clinical diagnostic laboratory services (CDLS). Serum ferritin was analysed at the Exeter clinical laboratory (Exeter, UK). HbA1c was measured with the Roche Cobas 6000 immunoassay, while Hemoglobin(Hb), Mean Corpuscular Volume(MCV), Mean Corpuscular Hemoglobin(MCH), and Mean Corpuscular Hemoglobin Concentration(MCHC) were measured by the Sysmex XN-1000^TM^ analyser.

### Statistical analysis

Categorical data were presented using frequencies and percentages, while continuous data were presented as medians [Inter-quartile range (IQR)] due to the nonparametric nature of most data. We compared HbA1c levels across different iron categories based on serum ferritin levels: category 1 (<58µg/L), category 2 (58 µg/L < 91 µg/L), category 3 (91 < 138 µg/L), category 4 (138 < 247 µg/L), and category 5 (≥ 247 µg/L). We then subdivided the participants into two groups based on serum ferritin concentration– iron-replete (≥ 70 µg/L) and iron-deplete (< 70 µg/L).

Differences between groups were assessed using the Chi-square test for categorical data and the Mann-Whitney U test for continuous data. Pearson’s correlation was used to evaluate the correlation between HbA1c and mean plasma glucose for both iron-replete and iron-deplete individuals. Additionally, a sensitivity analysis was conducted by matching iron-deplete and iron-replete individuals based on age and sex to control for potential confounding effects resulting from the initial large disparity in the case-to-control ratio. Linear regression analysis was used to assess the effect of iron deficiency (exposure) on HbA1c (outcome), adjusting for age, sex, duration of diabetes mellitus, history of hypertension, and body mass index (BMI) [11–15]. All statistical analyses were performed using Stata version 18.

### Ethical considerations

Ethical approval for the study was granted by the Research and Ethics committees of Uganda Virus Research Institute (UVRI-121/2019) and Uganda National Council of Science and Technology (UNCST) (HS 2588).

### Inclusivity in global research

Additional information regarding the ethical, cultural, and scientific considerations specific to inclusivity in global research is included in the Supporting Information (S1 Checklist).

## Results

### Baseline and clinical characteristics of participants

**Fig 1:**
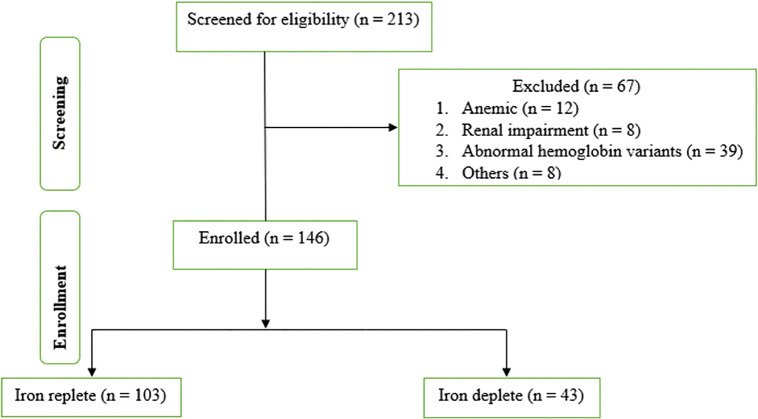
Participant flow chart.

A total of 146 participants were included in the study (Fig 1). Of these, 43 (29.5%) participants were classified as iron-deplete, while 103 (70.6%) were iron-replete. The prevalence of iron deficiency in rural areas was 22.9% and 42.2% among males and females, respectively (S1 graph). In urban areas, the proportions were lower, at 12.5% of males and 31.0% for females (S1 graph). No significant differences were observed between the iron-deplete and iron-replete groups concerning median age (55: IQR [50, 62] vs. 55 [45, 60], p = 0.323), BMI (27.3 [24.3, 30.2] vs. 27.1 [24.4, 30.7], p = 0.812), or duration of diabetes (Table 1).

**Table 1:** Baseline characteristics of the participants by iron deficiency status.

Variable	Iron replete n = 102	Iron deplete n = 43	p-value
Iron status (%)	70.6	29.5	
Female, sex n (%)	55(53.4)	32 (74.4)	0.029*
Age, years Median (IQR)	55 (50, 62)	55 (45, 60)	0.323**
BMI, Kg/m^2^	27.3 (24.3, 30.2)	27.1 (24.4, 30.7)	0.812*
DM duration, years	6 (3, 10)	6 (3, 10)	0.763*
**Glycaemia**			
HbA1c (mmol/mol)	69 (51.0, 85.0)	62 (46.0, 83.0)	0.291*
Fasting plasma glucose (mmol/L)	8.32 (6.1, 11.4)	8.1 (5.7, 10.4)	0.256*
Random plasma glucose (mmol/L)	13.2 (9.0, 17.2)	14.3 (8.3, 17.0)	0.899*
**Hematological indices**			
Hb (g/dL)	14.4 (13.8, 15.3)	14.1 (13.1, 14.8)	0.018*
MCV (fL)	89.7 (85.4, 93.0)	88.4 (86.5, 91.0)	0.991*
HCT (%) MCH (pg)	45.4 (43.1, 47.8) 28.5 (27.2, 29.7)	44.5 (42.9, 46.6) 28.2 (26.6, 29.4)	0.177* 0.295*
MCHC (g/dL)	32.0 (30.9, 33.0)	31.5 (30.2, 32.4)	0.030*

Table 1 shows the baseline characteristics of the participants. IQR: Interquartile range, BMI: Body Mass Index, DM: Diabetes Mellitus, HbA1c: Glycated haemoglobin, Hb: Haemoglobin, MCV: Mean Corpuscular Volume, HCT: Haematocrit, MCH: Mean Corpuscular Haemoglobin, MCHC: Mean Corpuscular Haemoglobin Concentration.

### Erythrocytic indices but not glycaemic measures differed between iron-replete and iron-deplete individuals

The concentration of haemoglobin (Hb) was lower among iron-deficient participants compared to iron-replete individuals (14.4 g/dl [13.8, 15.3] vs 14.1 [13.1, 14.8], P = 0.018) (Table 1). The mean corpuscular haemoglobin concentration (MCHC) was also lower among iron-deficient participants compared to iron-replete individuals (32.0 g/dl [30.9, 33.0] vs 31.5 [30.2, 32.4], P = 0.030). However, no significant differences were found in other indices, including Mean Cell Volume (MCV), Mean Corpuscular Haemoglobin (MCH), and haematocrit (HCT), between the two groups.

**Fig 2:**
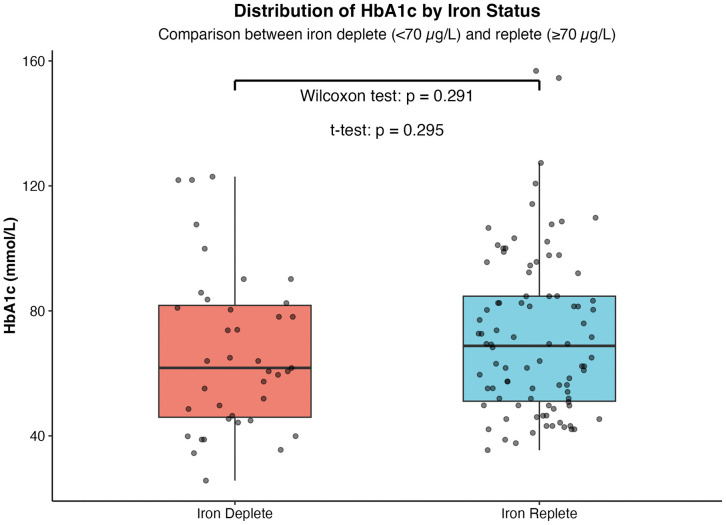
HbA1c distribution by iron status. Distribution of HbA1c by iron status (iron-deplete (<70 μg/L) vs iron-replete (≥70 μg/L) groups) (Wilcoxon test: p = 0.291).

In contrast, there was no difference in HbA1c distribution by iron status (iron-deplete (<70 μg/L) vs iron-replete (≥70 μg/L) groups) (Wilcoxon test: p = 0.291) (Fig 2). HbA1c levels were 69 [51.0, 85.0] vs 62 [46.0, 83.0] mmol/mol (P = 0.291). Other measures of glycaemic burden did not differ significantly between iron-replete and iron-deplete individuals: fasting glucose (8.32 [6.1, 11.4] vs 8.1 [5.7, 10.4] mmol/L, P 0.256), and random glucose (13.2 [9.0, 17.2] vs 14.3 [8.3, 17.0] mmol/L, P = 0.899). Furthermore, HbA1c levels did not differ significantly between the iron-deplete cases and their matched iron-replete controls (70.5 [55, 97] vs 64.0 [46.0, 80] mmol/mol, P = 0.05).

**Fig 3:**
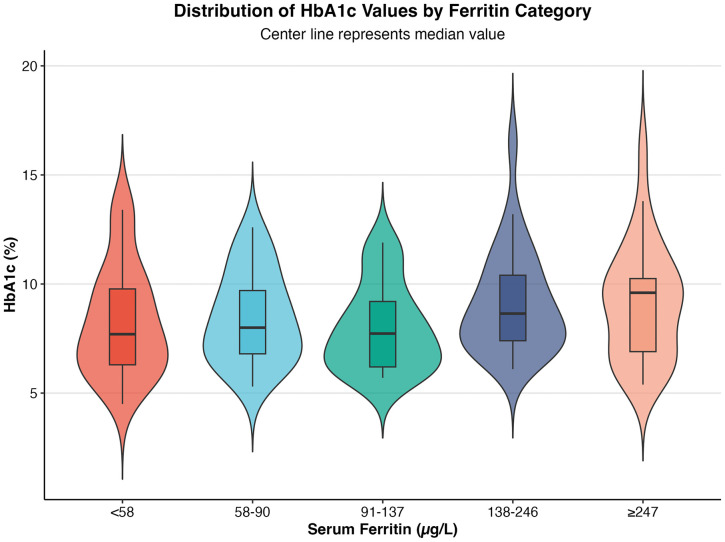
HbA1c distribution across iron categories. Mean of HbA1c across iron categories based on serum ferritin levels; category 1 (<58µg/L), category 2 (58 µg/L < 91 µg/L), category 3 (91 < 138 µg/L), category 4 (138 < 247 µg/L), category 5 (≥ 247 µg/L).

HbA1c distribution across five ferritin categories (<58, 58–90, 91–137, 138–246, ≥ 247 μg/L) showed an overlap between categories observed (Fig 3). Density distribution analysis further confirmed the substantial overlap between iron-deplete and iron-replete groups, with the iron-replete group showing slightly higher density in the 80–120 mmol/L range (S2 graph).

### Correlation between HbA1c with CGM glucose in both iron-deplete and replete participants

**Fig 4:**
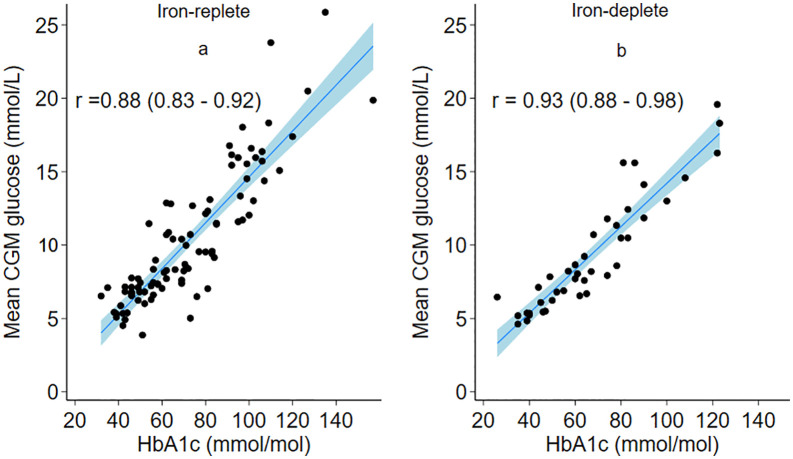
Correlation between HbA1c and CGM glucose.

There was a strong positive correlation between HbA1c and mean blood glucose for both iron-replete and iron-deplete individuals (Figs 4a and 4b). The association was similar among iron-deplete participants compared to iron-replete individuals. A stronger correlation between HbA1c and mean plasma glucose levels was observed in the iron-deplete cases compared to the iron-replete controls (Pearson Correlation coefficient: 0.93 (0.88–0.98) and 0.88 (0.83–0.92), respectively). A strong positive correlation between HbA1c (mmol/mmol) and mean continuous glucose was also observed between both the iron-replete controls (a) and their matched iron-deplete cases (b), as observed in the S3 graph. (Pearson Correlation coefficient: 0.83 (0.76–0.91) and 0.93 (0.89–0.98), respectively).

Scatterplots of the relationship between mean glucose obtained through continuous glucose monitoring and HbA1c in type 2 diabetes patients. Correlation between mean CGM and HbA1c in iron replete/normal iron state (Fig 4a) and iron-deplete state but without anaemia (Fig 4b). A solid straight line denotes the line of best fit, and the side lines represent the 95% confidence interval. Pearson’s correlation coefficient (r) and 95% confidence intervals (CI) are shown for each graph.

### Correlation between HbA1c with serum ferritin in both iron-deplete and iron-replete participants

The relationship between HbA1c and serum ferritin showed a weak positive correlation, as indicated by the median regression line (Scatter plot). However, substantial data point dispersion around this regression line indicated high variability (S4 graph).

### Effect of iron deficiency on HbA1c levels

From the multivariable model (Table 2), iron deficiency had no significant effect on HbA1c levels (P = 0.101). Hypertension and prolonged diabetes duration were associated with lower (−1.38%, 95% CI; −2.28 to −0.48, P = 0.003) and higher (0.15%, 95% CI; 0.07 to 0.24, P < 0.001) HbA1c levels, respectively. Age had an inverse association with HbA1c (−0.06%, 95% CI; −0.11 to −0.02, P = 0.003). Sex and BMI were not associated with significant changes in HbA1c levels.

**Table 2:** A linear regression model assessing the effect of the exposure (iron deficiency) against the outcome (HbA1c levels), adjusting for other a priori confounders.

Variable	Crude estimate (β) (95% CI)	P value	Adjusted estimate (β) (95% CI)	P value
**Iron deficiency**	−0.47 (−1.36, 0.42)	0.297	−0.72 (−1.58, 0.14)	0.101
**Age**	−0.05 (−0.09, −0.01)	0.014	−0.06 (−0.11, −0.02)	0.003
**Gender**				
Female	Reference			
Male	−0.20 (−1.03, 0.63)	0.635	−0.17 (−1.07, 0.73)	0.710
**BMI Categorised**				
Normal	Reference			
Underweight	1.15 (−1.31, 3.61)	0.358	1.54 (−0.75, 3.84)	0.185
Overweight	−0.53 (−1.57, 0.52)	0.323	−0.54 (−1.5, 0.45)	0.283
Obese	0.02 (−1.05, 1.09)	0.973	0.48 (−0.60, 1.56)	0.385
**Diabetes Mellitus duration**	0.04 (−0.03, 0.11)	0.247	0.15 (0.07, 0.24)	< 0.001
**History of hypertension**				
No	Reference			
Yes	−1.07 (−1.90, −0.23)	0.013	−1.38 (−2.28, −0.48)	0.003

Linear regression model adjusted for Age, gender, BMI, Diabetes Mellitus duration, and History of hypertension.

## Discussion

In this study involving non-anaemic participants with Type 2 diabetes mellitus (T2DM), we found no significant differences in mean HbA1c concentrations between iron-replete and iron-deplete individuals, and this result did not differ after adjusting for potential confounders, i.e., age, gender, BMI, hypertension status, and diabetes duration. Furthermore, we observed a strong positive correlation between HbA1c and mean CGM glucose in both groups, indicating that HbA1c is a dependable marker for monitoring glycaemic control in non-anaemic individuals with iron deficiency.

Similar results have been observed elsewhere by Heyningen et al. [16], Ford et al. [17] and Rai et al. [18] reporting no significant differences in HbA1c levels between iron-replete and iron-deficient individuals. The strong positive correlation observed between HbA1c and mean plasma glucose levels in our study is consistent with previous reports that relied only on single-point fasting glucose to estimate glycaemic exposure [19–21]. In another study, Hashimoto and Koga [22] observed a similar result among non-pregnant women; however, HbA1c levels significantly differed among the pregnant women. Other studies have reported contrasting results that iron deficiency impacts HbA1c levels amongst individuals [23]. Most of these studies, however, included methodological limitations, including small sample sizes and inadequate control for confounding factors such as anaemia and hemoglobinopathies.

Our findings that iron deficiency does not significantly alter HbA1c levels in non-anaemic type 2 diabetes patients warrants further investigation into the complex interplay between iron deficiency and HbA1c. Iron is a crucial precursor in erythropoiesis [24], playing a key role in the formation of new red blood cells(RBCs). In iron deficiency, this reduced availability of iron reduces the production of new RBCs, resulting in a higher proportion of older RBCs, which have been more exposed to glycaemia. The resulting predominance of older, more glycated cells may offset any reduction in HbA1c expected from impaired haemoglobin synthesis. Consequently, when iron stores are replenished during iron replacement therapy, the erythropoietic process is restored, and the influx of younger, less glycated erythrocytes leads to a modest fall in HbA1c, as previously reported [25].

This study has clinical implications, given the global burden of iron deficiency [26]. The diagnosis of iron deficiency without anaemia remains diagnostically challenging, requiring specialised assays that are often unavailable in peripheral healthcare settings [27]. Our findings suggest that in non-anaemic patients with T2D, iron deficiency does not significantly alter HbA1c levels, supporting its continued reliability as a marker of long-term glycaemic control.

However, this study has some limitations that should be taken into consideration when interpreting findings. First, iron status was assessed using serum ferritin, a widely used but inflammation-sensitive marker of iron stores [28,29]. Given the low-grade inflammation typical of diabetes, ferritin may overestimate true iron stores. Future studies should incorporate inflammation-independent biomarkers, such as soluble transferrin receptor (sTfR), for more accurate classification [30]. Second, because this was a secondary data analysis of the OPTIMAL dataset, the range of available variables was limited. We were unable to determine the underlying causes of iron deficiency (e.g., chronic blood loss versus decreased iron absorption) and do subgroup analyses to assess the impact of the different underlying causes of iron deficiency [27]. HbA1c reflects glycaemic exposure over 90−120 days, far exceeding the 14-day duration of the original study period [31]. Finally, we did not screen for other red-cell disorders such as glucose-6-phosphate dehydrogenase (G6PD) variants [32].

Evidence remains limited on how iron deficiency, independent of anaemia, impacts HbA1c, as most studies reporting conflicting results have focused on iron deficiency anaemia rather than isolated iron depletion [17,33–36].

In conclusion, our findings indicate that HbA1c strongly correlates with mean blood glucose levels, even in iron-depleted states, among non-anaemic individuals with T2DM, supporting its reliability as a marker of glycaemic control. However, larger studies are needed to confirm these observations, and delineate their implications for screening and diagnostic purposes.

## Supporting information

S1 Graph Prevalence of iron deficiency (ID) by (a) site and by (b) site and sex (grey; female and white; male). (TIF)

S2 Graph Density distribution across five ferritin categories (<58, 58–90, 91–137, 138–246, ≥ 247 μg/L). (TIF)

S3 Graph Correlation between HbA1c (mmol/mmol) and mean continuous glucose among iron-deplete cases (b) and their matched iron-replete controls (a). A solid straight line denotes the line of best fit, and the side lines represent the 95% confidence interval. Pearson’s correlation coefficient (r) and 95% confidence intervals are shown for each graph. (TIF)

S4 Graph Correlation between HbA1c with serum ferritin in both iron-deplete and iron-replete participants. (TIF)

S1 Checklist Inclusivity-in-global-research-questionnaire. (DOCX)

S1 Dataset Real.csv A dataset containing all the variables used in the analysis. (CSV)
